# Insecticidal effects of some selected plant extracts against *Anopheles stephensi* (Culicidae: Diptera)

**DOI:** 10.1186/s12936-022-04320-5

**Published:** 2022-10-21

**Authors:** Merdya Muhammed, Sisay Dugassa, Merga Belina, Sarah Zohdy, Seth R. Irish, Araya Gebresilassie

**Affiliations:** 1 Biology Department, College of Natural and Computational Sciences, Jinka University, Jinka, Ethiopia; 2 Aklilu Lemma Institute of Pathobiology, Addis Ababa University, Addis Ababa, Ethiopia; 3 Department of Statistics, College of Natural and Computational Sciences, Addis Ababa University, Addis Ababa, Ethiopia; 4 Division of Epidemiology and Biostatistics, Department of Global Health, Faculty of Medicine and Health Sciences, Stellenbosch University, Western Cape, South Africa; 5 US President’s Malaria Initiative, Entomology Branch, Centers for Disease Control and Prevention, GA Atlanta, USA; 6 Department of Zoological Sciences, College of Natural and Computational Sciences, Addis Ababa University, Addis Ababa, Ethiopia

**Keywords:** Adulticide, *Anopheles stephensi*, Botanical, Larvicide, Plant extract

## Abstract

**Background:**

The use of synthetic insecticides against mosquitoes may lead to resistance development and potential health hazards in humans and the environment. Consequently, a paradigm needs to shift towards the alternative use of botanical insecticides that could strengthen an insecticide resistance management programme. This study aimed to assess the insecticidal effects aqueous, hexane, and methanol crude leaf extracts of *Calpurnia aurea*, *Momordica foetida*, and *Zehneria scabra* on an insectary colony of *Anopheles stephensi* larvae and adults.

**Methods:**

Fresh leaves of *C*. *aurea*, *M*. *foetida* and *Z*. *scabra* were collected and dried, then separately ground to powder. Powdered leaves of test plants were extracted using sonication with aqueous, hexane, and methanol solvents. The extracts were concentrated, and a stock solution was prepared. For comparison, Temephos (Abate®) and control solutions (a mixture of water and emulsifier) were used as the positive and negative controls, respectively. Different test concentrations for the larvae and the adults were prepared and tested according to WHO (2005) and CDC (2010) guidelines to determine lethal concentration (LC) values. Mortality was observed after 24 h exposure. The statistical analyses were performed using Statistical Package for the Social Sciences (SPSS) software (Kruskal-Wallis test) and R software (a generalized linear model was used to determine LC_50_ and LC_90_ values of the extracts).

**Results:**

The lowest LC_50_ values were observed in aqueous extracts of *M*. *foetida* followed by *Z*. *scabra* extract and *C*. *aurea* leaves at 34.61, 35.85, and 38.69 ppm, respectively, against the larvae. Larval mortality was not observed from the hexane extracts and negative control, while the standard larvicide (temephos) achieved 100% mortality. Further, the adulticidal efficacy was greatest for aqueous extract of *Z*. *scabra* with LC_50_ = 176.20 ppm followed by aqueous extract of *C*. *aurea* (LC_50_ = 297.75 ppm).

**Conclusion:**

The results suggest that the leaf extracts of the three test plants have the potential of being used for the control of vector *An*. *stephensi* larvae and adult instead of synthetic mosquitocides. Further studies need to be conducted to identify the active ingredients and their mode of action.

## Background

Mosquitoes are among the most important groups of arthropods with medical significance. They transmit several important parasitic and arboviral diseases, such as malaria, filariasis, dengue, yellow fever, and Rift Valley fever [1]. Malaria, caused by protozoans (*Plasmodium* spp.), can lead to high mortality and morbidity [2]. In 2019 there were 229 million reported malaria cases [2]. Malaria cases that occurred in 2020 were estimated to be 241 million, with 627,000 deaths reported from 85 countries. Around 95% of the malaria cases and 96% of malaria deaths were found in sub-Saharan Africa, with 80% of all malaria deaths in Africa estimated to be among children under the age of five [3].

*Anopheles stephensi* is a major malaria vector in South Asia and the Middle East, including the Arabian Peninsula [4], and is known to transmit both the major human malaria parasites *Plasmodium falciparum* and *Plasmodium vivax* [5]. In 2012, *An. stephensi* was first reported from the Horn of Africa as an invasive species in Djibouti. In 2016 and 2019, it was reported from additional countries, including Ethiopia, Somalia, and the Republic of Sudan [6–8]. If the species continues to spread across the continent, it is estimated that an additional 126 million people in Africa will be at risk of malaria [9]. In Ethiopia, this malaria vector was first reported from the Somali region in 2016 [10] and was later found to be widely present in urban areas of northeastern Ethiopia [11, 12]. The spread of this vector in different parts of the country has become a serious concern for malaria prevention and elimination strategies. The World Health Organization (WHO) has issued a warning about the invasion and spread of *An*. *stephensi* particularly in urging African national malaria control programmes and their partners to be vigilant in areas of risk and to improve and enhance their monitoring systems to identify and control this invasive mosquito species [13]. Recent evidence reveals that invasive *An*. *stephensi* from Ethiopia is a more competent vector for *P*. *vivax* than *Anopheles arabiensis*, the primary malaria vector in Ethiopia, in laboratory experiments [14].

Global malaria cases and deaths have been significantly reduced following the scaling up of long-lasting insecticidal nets (LLINs) and indoor residual spraying (IRS) [15, 16]. However, widespread use of synthetic insecticides in controlling mosquito vectors has resulted in the persistence and accumulation of non-biodegradable chemicals in the ecosystem, development of resistance to insecticides in vectors, and toxic effects in non-target organisms [17–19]. As evidenced in recent studies from different parts of Ethiopia, *An*. *arabiensis* and *An*. *stephensi* have shown resistance to insecticides belonging to four of the chemical classes approved for IRS and ITNs, including DDT (organochlorine), malathion (organophosphate), bendiocarb and propoxur (carbamates), and alpha-cypermethrin, cyfluthrin, deltamethrin, etofenprox, lambda-cyhalothrin, and permethrin (pyrethroids) [20–23]. Reduced effectiveness of insecticides on these malaria vectors may aid in the invasion and establishment of *An. stephensi*.

The emergence of insecticide resistance necessitates an urgent need to develop new and improved mosquito control methods that are economical and effective and less toxic to non-target organisms and the environment. In this regard, botanicals, namely plant extracts and essential oils with insecticidal potential, are recognized as potent alternatives to replace the synthetic insecticides in mosquito control programmes due to their larvicidal, pupicidal, and adulticidal properties; these have also been shown to have oviposition inhibiting, repellent or insect growth regulatory effects, and may help us to find chemicals that are safe, biodegradable, and target specific [24–27].

Traditionally, plant-based products have constituted an important source of insecticides and other pharmaceutical drugs for many centuries; *Calpurnia aurea*, *Momordica foetida*, and *Zehneria scabra* are the foremost mentioned in Africa [28, 29]. In Ethiopia, these botanicals have been reported as having medicinal properties to prevent vector-borne diseases [30–32] and protect against insect pests [33, 34]. Moreover, these three medicinal plants are cheap and easily available. However, the bioactivities of these plant extract against invasive *An*. *stephensi* in Ethiopia has not been evaluated yet. This study evaluated the larvicidal and adulticidal activities of the aforementioned plant leaf extracts against *An*. *stephensi* under laboratory conditions.

### Methods

#### Collection of plant samples

The fully developed fresh leaves of *C*. *aurea* and *M*. *foetida* were collected around Bahir Dar University campus located at 11°34′28.0″N, 37°23′53.4″E, at an altitude of 1801 m, while the leaves of *Z*. *scabra* were collected from Akaki District, Addis Ababa (8°49′40.5″N, 38°50′23.6″E, altitude 2117 m). The plant species were authenticated by a plant taxonomist from the National Herbarium in the Department of Plant Biology and Biodiversity Management, Addis Ababa University. The voucher specimens (MM-01 of *M*. *foetida*, MM-02 of *C*. *aurea*, and MM-03 of *Z*. *scabra*, respectively) were deposited at the National Herbarium, College of Natural and Computational Sciences of Addis Ababa University, Ethiopia.

### Preparation of plant samples

The leaves were washed with water and air-dried separately under shade at room temperature for two weeks in the Insect Sciences Laboratory, Addis Ababa University. Finally, the dried leaves were manually ground by pestle and mortar through a sieve into a fine powder. The leaf powder was kept at room temperature in labeled air-tight plastic bags until used.

### Extraction of the plant samples

The crude extraction processes were conducted in the Organic Chemistry Laboratory of the Department of Chemistry, Addis Ababa University. Seventy-two grams of each powdered material was soaked separately in 720 ml of hexane, methanol, and distilled water at a ratio of 1:10 (W/V) in Erlenmeyer flask. Afterward, the mixtures were sonicated by ultrasonic cleaner apparatus (USC-T, Malaysia) at 20 kHz frequency with 11 W power twice per day for 15 min for two days. The mixtures were left to settle for 10 min and then cooled at room temperature. The supernatant of the hexane and methanol crude extracts were filtered through Whatman No.3 (Whatman International Ltd., Maid stone, England) filter paper, while the aqueous extracts using suction were filtered through a Buchner funnel with Whatman filter paper No.1. The filtrates of hexane and methanol crude extracts were concentrated using a vacuum rotary evaporator (Rota-vapor-RE, Buchi Labortechnik AG, Flawil, Switzerland) under reduced pressure at 40^o^C, while the aqueous crude extracts were evaporated to dryness using a lyophilizer. The residue obtained from each plant extract was left to cool at room temperature to remove traces of solvent, and then finally, were collected separately in a Wheaton bottle glass containers and were preserved a refrigerated at 4 °C until used for experimentation.

### Rearing of *Anopheles stephensi*

Larvicidal and adulticidal bioassays were conducted with colony larvae and adults of *An*. *stephensi* originated from the Awash Arba area, eastern Ethiopia. The mosquito larvae and adults were maintained at 28 ± 3^o^C temperature with 70 ± 10% relative humidity at the insectary of Aklilu Lemma Institute of Pathobiology, Addis Ababa University. All equipment (cages, trays, incubators) containing mosquitoes have been deployed so that accidental contact and release was minimized.

The larvae (67 larvae/cm^2^) were kept in plastic trays (20 cm × 15 cm × 5 cm) containing de-chlorinated water (1.5 l) and were maintained under 12:12 h light and dark photoperiod cycles in the laboratory following the standard mosquito rearing procedure [35]. The larvae were fed with yeast (Saf-Instant yeast). The media were changed every three days. The pupae formed were collected by a glass beaker and transferred to 24.5 × 24.5 × 24.5 cm cages (Bug dorms) for adult emergence. Newly emerged adults were maintained in mosquito cages at 28 ± 3 °C temperature and 70 ± 10% relative humidity and fed sterile 10% sugar solution soaked in cotton pads within Petri dishes. The cotton was always kept moist with the solution and changed every day. In addition to sugar feeding, female mosquitoes were allowed to take blood meals from a restrained rabbit three times a week for egg development and oviposition. Moist filter papers in cups were placed inside rearing cages for oviposition by gravid female mosquitoes. The eggs were washed off with deionized water onto larval rearing trays and allowed to hatch into neonate larvae in the laboratory. Third to fourth instar larvae and bloodmeal starved 2 to 5 days old adult female *An*. *stephensi* were used continuously for larvicidal and adulticidal tests, respectively.

### Preparation of stock solution

One gram of each crude plant extract was placed separately in 250 ml Wheaton glass bottles and dissolved in 100 ml of distilled water for methanol and aqueous extracts, while the hexane extracts were first dissolved in 4 ml of acetone and then added to 96 ml of distilled water to prepare 100 ml of a 1% stock solution. In the stock solution, one drop of emulsifier (Tween 80) was added to each extract at a concentration of 0.05%. From the 1% stock solution, concentrations of 25, 50, 100, 150, 200, 250, and 300 ppm for the larvae, and concentrations of 20, 40, 80, 160, and 320 ppm for the adult were prepared for exposure of the target mosquito.

### Larvicidal bioassay

Larvicidal activities of each leaf crude extract were measured according to WHO standard procedures [35]. For larvicidal bioassay, hexane, methanol and aqueous crude leaf extracts of the test plants were screened at 25, 50, 100, 150, 200, 250, and 300 ppm of test concentrations. Twenty individuals of late third to early fourth instar larvae were kept in a 300 ml enamel cups containing 99 ml of distilled water and 1-ml of desired concentrations of crude solvent extracts of *C*. *aurea*, *M*. *foetida* and *Z*. *scabra* were added. In the same way, 99 ml of distilled water with 1 ml mixture of acetone (for hexane extracts) and Tween 80 were made up to 100 ml to serve as a negative control. In addition, temephos at the rate of 0.25 ppm within a similar volume of test cups was used as a positive control [36]. The experiment was conducted for 24 h at the optimum conditions of 28 ± 3 °C temperature and 70 ± 10% relative humidity under 12:12 light and dark photoperiod in the insectary. During the exposure period, no food was offered to the larvae. Each experiment was run three times on different days along randomly set up with appropriate negative control and standard check. Numbers of dead larvae were counted after 24 h of exposure, and the percentage of mortality was reported from the averages of twelve replicates. The dead larvae included moribunds; those incapable of rising to the surface in each concentration of treatments, and the standards were combined separately and expressed as the average mortality to determine LC_50_ and LC_90_ values.

### Adulticidal bioassay

Adulticidal activities of each crude solvent extract were performed using the CDC bottle bioassays [37, 38]. Most of the synthetic insecticides available for the control of adult mosquitoes, in particular *An*. *stephensi* were reported to be resisted by adults [12, 23], thus, this bioassay did not have any synthetic insecticide as a positive control. In this bioassay, aqueous, hexane and methanol crude leaf extracts of the three plants were screened at 25, 50, 100, 150, 200, 250, and 300 ppm concentrations. Two hundred fifty ml Wheaton bottles with screw lids were properly cleaned and dried, and then they were coated with 1ml solution of the desired concentrations of the tested extracts by swirling assuring complete coating of the bottle and its cap. Similarly, 1ml of acetone and emulsifier (Tween 80) solution was added to the control bottle handled as before.

Adult mosquitoes (20, aged 2–5 days) fed on 10% sucrose solution were released to each bottle, including the control bottle with the help of mouth aspirator. The opening of each bottle was closed with their lids after introduction of mosquito adults. The exposure time was set to 2 h. After that, the mosquitoes were transferred into 250 ml capacity plastic cups using aspirator, where they were provided with 10% sucrose solution and the mortalities were checked after 24 h. The experiment was done at 28 ± 3 °C temperature and 70 ± 10% relative humidity under 12:12 light and dark photoperiod in the insectary. The percentage of adult mortality was corrected using Abbott’s formula [39] when needed.

$$\% \text{Morality} = \frac{{\% \text{test}\,\text{morality} - \% \text{control}\, \text{morality}}}{{100 - \% \text{control}\, \text{morality}}} \times 100. $$

### Data analysis

The mean percentage mortality was analysed using SPSS version 25.0 software (SPSS Inc, Chicago, IL, USA), and dose-dependent mortality also was performed by R software version 4.0.5. The percentage mortality of larvae and adult were checked for normality by 1-Sample Kolmogorov-Smirnov Z test (K-S). When the percent mortality did not conform to the normal distribution, the non-parametric equivalent tests of Kruskal-Wallis were followed. p-values were adjusted with the Bonferroni correction to adjust for the inflation of type I errors when several Mann–Whitney tests are performed [40]. The LC_50_ and LC_90_ values were calculated using a generalized linear probit model, considering a 95% confidence interval.

## Results

### Larvicidal activity of plant extracts against *An*. *stephensi*

Larval mortalities obtained from bioassays of aqueous and methanol extracts of *Calpurnia aurea*, *Momordica foetida*, and *Zehneria scabra* leaves against *An*. *stephensi* larvae after 24 h exposure periods are presented in Tables 1, 2 and 3. All aqueous and methanol test plant extracts showed low, moderate, and high larvicidal activities tested between 25 ppm-300 ppm treatments against the late 3rd to early 4th instar larvae of *An*. *stephensi*. Within the same exposure period, larval mortality was not observed for the tests using hexane crude leaf extracts and the negative control. The standard positive control (temephos) achieved 100% larval mortality.

**Table 1:** Mean % larval mortality of *Calpurnia aurea* crude leaf solvent extracts at different rates against the late 3rd and early 4th instar *Anopheles stephensi* larvae after 24 h of exposure

Concentration(ppm)	% Mean mortality ± SE	
	Solvents	
	Aqueous	Methanol
25	35.00 ± 2.6^Aa^	23.75 ± 3.65^Aa^
50	60.00 ± 3.54^Ba^	35.00 ± 0.00^Bb^
100	86.25 ± 2.23^Ca^	90.42 ± 1.4^Ca^
150	99.58 ± 0.42^Da^	95.00 ± 2.46^DCa^
200	100.00 ± 0.00^Da^	96.67 ± 1.42^Da^
250	100.00 ± 0.00^Da^	100.00 ± 0.00^Da^
300	100.00 ± 0.00^Da^	100.00 ± 0.00^Da^
Temephos (0.25)	100.00 ± 0.00^Da^	100.00 ± 0.00^Da^
Control	0.00 ± 0.00^Ea^	0.00 ± 0.00^Ea^

*Each value of % mean mortality ± SE represents the value of twelve replicates

*Means values followed by the same letters across column (Upper case) and row (Lower case) are not statistically significantly different (Multiple-Mann Whitney U-test; p > 0.003)

There was a significant difference and interaction effects between all aqueous and methanol test plant extract against the tested larvae (Kruskal-Wallis test, p-value < 0.05). The mean percent larval mortality between treatments, control, and the standard groups had a statistically significant difference (Kruskal-Wallis test, p-value < 0.05). The mortality effect of the test plant extracts against *An*. *stephensi* larvae were dose and extraction solvent dependent.

In the *C. aurea* test plant, the highest (100%) larval mortality was found both in aqueous and methanol extracts at 200 ppm and 250 ppm of treatments, respectively (Table 1). Even at 150 ppm, the larvicidal effects of aqueous and methanol extracts of *C*. *aurea* were not significantly different from standard checks (Multiple-Mann Whitney U test, p-value > 0.003; Table 1). Yet, all *C*. *aurea* treatments significantly affected larval mortality compared to the negative control (Multiple-Mann Whitney U test; p < 0.003).

Aqueous and methanol extracts of the *M*. *foetida* test plant showed 100% larval mortality at 200 ppm and 250 ppm of treatments, respectively (Table 2). Moreover, larvicidal activity found from aqueous extracts of *M*. *foetida* at 100 ppm and methanol extracts at 200 ppm caused mortality (ranging 95–97.9%) was not significantly different from the mortality achieved in temephos exposed on *An*. *stephensi* larvae (Multiple-Mann Whitney U test, p-value > 0.003; Table 2). Statistical differences were observed between all *M*. *foetida* treatments and negative control (Multiple-Mann Whitney U test; p-value < 0.003).

**Table 2:** Mean % larval mortality of *Momordica foetida* crude leaf solvent extracts at different rates against the late 3rd and early 4th instar *Anopheles stephensi* larvae after 24 h of exposure

Concentration(ppm)	% Mean mortality ± SE	
	Solvents	
	Aqueous	Methanol
25	27.5 ± 2.85^Aa^	4.55 ± 1.4^Ab^
50	80.83 ± 4.51^Ba^	20.83 ± 2.53^Bb^
100	95.00 ± 1.74^Ca^	45.83 ± 2.20^Cb^
150	97.92 ± 0.96^Ca^	71.67 ± 3.39^Db^
200	100.00 ± 0.00^Ca^	97.5 ± 2.50^Ea^
250	100.00 ± 0.00^Ca^	100.00 ± 0.00^Ea^
300	100.00 ± 0.00^Ca^	100.00 ± 0.00^Ea^
Temephos (0.25)	100.00 ± 0.00^Ca^	100.00 ± 0.00^Ea^
Control	0.00 ± 0.00^Da^	0.00 ± 0.00^Fa^

*Each value of % mean mortality ± SE represents the value of twelve replicates

*Means values followed by the same letters across column (Upper case) and row (Lower case) are not statistically significantly different (Multiple-Mann Whitney U-test; p-value > 0.003)

**Table 3:** Mean % larval mortality of *Zehneria scabra* crude leaf solvent extracts at different rates against the late 3rd and early 4th instar *Anopheles stephensi* larvae after 24 h of exposure

Concentration(ppm)	% Mean mortality ± SE	
	Solvents	
	Aqueous	Methanol
25	29.17 ± 3.68^Aa^	23.75 ± 2.69^Aa^
50	73.33 ± 3.9^Ba^	66.25 ± 5.04^Ba^
100	95.42 ± 1.44^Ca^	87.08 ± 2.17^Ca^
150	97.08 ± 1.44^Ca^	92.50 ± 1.44^Da^
200	99.50 ± 0.42^Ca^	99.17 ± 0.83^Ea^
250	100.00 ± 0.00^Ca^	100.00 ± 0.00^Ea^
300	100.00 ± 0.00^Ca^	100.00 ± 0.00^Ea^
Temephos (0.25)	100.00 ± 0.00^Ca^	100.00 ± 0.00^Ea^
Control	0.00 ± 0.00^Da^	0.00 ± 0.00^Fa^

*Each value of % mean mortality ± SE represents the value of twelve replicates

*Means values followed by the same letters across column (Upper case) and row (Lower case) are not statistically significantly different (Multiple-Mann Whitney U-test; p-value > 0.003)

Among the two solvent test concentrations of *Z*. *scabra* extracts, statistically, non-significant differences were observed (Multiple-Mann Whitney U test, p-value > 0.003; Table 3). However, their larvicidal activity was significantly different from negative control (Multiple-Mann Whitney U test; p-value < 0.003). At the highest concentrations (250 ppm), both aqueous and methanol *Z*. *scabra*, crude leaf extracts resulted in 100% larval mortality (Table 3). Moreover, aqueous extracts of *Z*. *scabra* at 100 ppm, and methanol extracts at 200 ppm, each caused more than 95% larval mortality, had no significant effect compared to mortality achieved in temephos treatment (Multiple-Mann Whitney U test; p-value > 0.003;).

The lethal toxicity doses of aqueous and methanol crude leaf extracts of *C*. *aurea*, *M*. *foetida*, and *Z*. *scabra* against larvae of *An. stephensi* are shown in Table 4. Crude aqueous leaf extract of *M*. *foetida* showed strong larvicidal activity, having an LC_50_ value of 34.61 ppm and LC_90_ value of 57.61 ppm, followed by *Z*. *scabra* (LC_50_ = 35.85 ppm; LC_90_ = 68.26 ppm) and *C*. *aurea* (LC_50_ = 38.69 ppm; LC_90_ = 108.28 ppm). Similarly, the methanol leaf extract of *Z*. *scabra* had good larvicidal activities with LC_50_ and LC_90_ values of 41.32 ppm and 99.07 ppm, followed by *C*. *aurea* (LC_50_ = 43.25 ppm; LC_90_ = 96.02 ppm). However, the crude methanol leaf extract of *M. foetida* had lower larvicidal toxicity against the larvae of *An. stephensi* compared to other test plant extracts (LC_50_ = 99.50 ppm; LC_90_ = 188.76 ppm, Table 4).

**Table 4:** LC_50_ and LC_90_ (ppm) of aqueous and methanolic crude leaf extracts of tests plants against the late third to early fourth instar larvae of *Anopheles stephensi*

Test plants	Solvents	LC_50_ (ppm)	95% Confidence limit		LC_90_ (ppm)	95% Confidence limit		χ2 (df = 5)
			LCL	UCL		LCL	UCL	
*C*. *aurea*	Aqueous	38. 69	35. 57	41.82	108. 28	96. 27	120. 30	2.93
	Methanol	43. 25	40. 49	46. 02	96. 02	83. 87	108. 18	4.15
*M*. *foetida*	Aqueous	34. 61	32. 84	36. 38	57. 61	53. 29	61. 93	0.71
	Methanol	99. 50	94. 56	104. 43	188. 76	177. 70	199. 82	6.90
*Z*. *scabra*	Aqueous	35. 85	33. 79	37. 91	68. 26	60. 20	76. 32	0.37
	Methanol	41. 32	38. 51	44. 12	99. 07	84. 64	113. 50	1.43

### The adulticidal activity of plant extracts against *An*. *stephensi*

Tables 5, 6 and 7 show the mean percentage mortalities and toxicity effects of adult *An*. *stephensi* after 24 h exposures to different solvent extracts of *C*. *aurea*, *M*. *foetida*, and *Z*. *scabra*. Statistically significant differences in mean percentage mortality among the different extracts and the negative control were observed (Kruskal-Wallis test, p < 0.05). The adulticidal data showed that the mortality rate of the vector was directly proportional to the concentration and extraction solvents. All hexane test plant extracts showed lower adulticidal property against *An*. *stephensi* (≤ 20% at all treatments tested in the bioassays, Tables 5, 6 and 7). Between the different solvent leaf extracts of *C. aurea*, only aqueous extract gave 55% adult mortality of *An. stephensi* at 320 ppm, while other crude solvent leaf extracts had lower adulticidal activity, having adult mean mortalities ranging from 0 to 48% (Table 5).

**Table 5:** Mean % adult mortality of *Calpurnia aurea* crude leaf solvent extracts at different treatments against *An*. *stephensi*

Concentration (ppm)	% Mean mortality ± SE		
	Solvents		
	Aqueous	Methanol	Hexane
20	1.67 ± 1.67^Aa^	0.00 ± 0.00^Aa^	0.00 ± 0.00^Aa^
40	6.67 ± 1.67^Aa^	6.67 ± 1.67^Ba^	0.00 ± 0.00^Ab^
80	16.67 ± 1.67^Ba^	16.67 ± 1.67^Ca^	0.00 ± 0.00^Ab^
160	21.67 ± 1.67^Ba^	26.67 ± 3.33^Da^	6.67 ± 3.33^Bb^
320	55.00 ± 5.77^Ca^	48.33 ± 1.67^Ea^	18.33 ± 1.67^Cb^
Control	0.00 ± 0.00^Da^	0.00 ± 0.00^Aa^	0.00 ± 0.00^Aa^

*Each value of % mean mortality ± SE represents the value of three replicates

*Means values followed by the same letters across column (Upper case) and row (Lower case) are not statistically significantly different (Multiple-Mann Whitney U-test; p-value > 0.003)

Different solvent (aqueous, methanol, and hexane) crude leaf extracts of *M*. *foetida* resulted in lower adult mortalities of *An*. *stephensi* (< 24%) at all treatments tested in the bioassays and were not effective against adults of malaria vector *An*. *stephensi* (Table 6).

**Table 6:** Mean % adult mortality of *Momordica foetida* crude leaf solvent extract at different concentrations against *Anopheles stephensi*

Concentration(ppm)	% Mean mortality ± SE		
	Solvents		
	Aqueous	Methanol	Hexane
20	0.00 ± 0.00^ABa^	0.00 ± 0.00^Aa^	0.00 ± 0.00^Aa^
40	1.67 ± 1.67^Ba^	0.00 ± 0.00^Aa^	0.00 ± 0.00^Ab^
80	5.00 ± 2.89^Ba^	1.67 ± 1.67^Ba^	0.00 ± 0.00^Ab^
160	13.33 ± 1.67^Ca^	13.33 ± 1.67^Ca^	0.00 ± 0.00^Ab^
320	18.33 ± 4.41^Ca^	23.33 ± 1.67^Da^	1.67 ± 1.67^Bb^
Control	0.00 ± 0.00^Aa^	0.00 ± 0.00^Aa^	0.00 ± 0.00^Aa^

*Each value of % mean mortality ± SE represents the value of three replicates

*Means values followed by the same letters across column (Upper case) and row (Lower case) are not statistically significantly different (Multiple-Mann Whitney U-test; p-value > 0.003)

There were statistically significant differences in mean percentage mortality among different concentrations of aqueous, methanol, and hexane leaf extracts of *Z*. *scabra* and with negative control (Kruskal-Wallis test; p-value < 0.05; Table 7). Within the given time intervals adult mortality was recorded for the control treatment. However, the highest adult mortality (84%) at higher concentration 320 ppm against *An*. *stephensi* was recorded in aqueous crude leaf extract of *Z*. *scabra* followed by its methanol extracts (65%) and their adulticidal potential was statistically different from the negative control (Multiple-Mann Whitney U-test; p-value < 0.003, Table 7).

**Table 7:** Mean % adult mortality of *Zehneria scabra* crude leaf solvent extracts at different concentrations against *Anopheles stephensi*

Concentration (ppm)	% Mean mortality ± SE		
	Solvents		
	Aqueous	Methanol	Hexane
20	4.33 ± 2.6^Aa^	11.67 ± 3.33^Aa^	0.00 ± 0.00^Aa^
40	11.67 ± 1.67^Ba^	20.00 ± 2.89^ABa^	0.00 ± 0.00^Ab^
80	26.67 ± 3.33^Ca^	23.33 ± 3.33^Ba^	0.00 ± 0.00^Ab^
160	36.67 ± 6.00^Ca^	41.66 ± 4.40^Ca^	15.00 ± 2.89^Bb^
320	84.00 ± 2.89^Da^	65.00 ± 5.77^Db^	20.00 ± 2.89^Bc^
Control	0.67 ± 0.45^Ea^	0.67 ± 0.45^Ea^	0.67 ± 0.45^Aa^

*Each value of % mean mortality ± SE represents the value of three replicates

*Means values followed by the same letters across column (Upper case) and row (Lower case) are not statistically significantly different (Multiple-Mann Whitney U-test; p-value > 0.003)

The lethal effects of different crude leaf extracts against adults of *An*. *stephensi* are shown in Table 8. All hexane and *M*. *foetida* crude leaf extracts had low adulticidal impacts, only aqueous and methanol *C*. *aurea* and *Z*. *scabra* leaf extracts were subjected to dose-response bioassay to detect the lethal concentrations. From the dose-response curves, the lowest LC_50_ (= 176.20 ppm and 205.41 ppm) and LC_90_ (= 425.13 and 883.11 ppm) values were demonstrated from the aqueous and methanol *Z*. *scabra* crude leaf extracts, respectively (Table 8), and its relative toxicities against adult *An*. *stephensi* was much higher than the rest of the plant extracts. The highest LC_50_ (= 297.75 ppm and 333.27 ppm) and LC_90_ (= 762.63 ppm and 1031.74 ppm) values were from the aqueous and methanol *C*. *aurea* crude leaf extracts, respectively, and its relative toxicity on *An*. *stephensi* was much lower than *Z*. *scabra* crude extracts.

**Table 8:** LC_50_ and LC_90_ (ppm) of aqueous and methanolic crude leaf extracts of *Calpurnia aurea* and *Zehneria scabra* against adults of *Anopheles stephensi*

Test plants	Solvents	LC_50_	95% Confidence limit		LC_90_	95% Confidence limit		χ2 (df = 3)
			LCL	UCL		LCL	UCL	
*C*. *aurea*	Aqueous	297. 75	254. 95	340. 56	762. 63	491. 13	1034. 13	0.88
	Methanol	333. 27	268. 92	397. 63	1031. 74	569. 06	1494. 43	0.75
*Z*. *scabra*	Aqueous	176.20	157. 82	194. 58	425. 13	346. 14	504. 12	2.70
	Methanol	205. 41	172. 29	238. 54	883. 11	529. 33	1236. 90	0.70

## Discussion

Synthetic insecticides play a vital role in insect vector control programmes. However, due to environmental concerns and the development of resistance to synthetic insecticides, there is a growing effort to explore plants to obtain bioactive compounds that are safe for non-target animals and do not pose residue problems, but are still able to suppress vector populations. Here, the present study evaluated the larvicidal and adulticidal activities of crude leaf solvent extracts of *C*. *aurea*, *M*. *foetida*, and *Z*. *scabra*, native plants to Ethiopia, which were tested against *An*. *stephensi*, an invasive malaria vector in Ethiopia. In this study, all hexane crude leaf extracts showed ineffectual insecticidal activity on *An*. *stephensi* larvae and adults. However, aqueous and methanol extracts of the test plants gave promising results to be used as mosquitocides. This could suggest the presence of more polar solvent-soluble phytochemicals in leaves of *C*. *aurea*, *M*. *foetida*, and *Z*. *scabra*, which could be responsible for the observed bioactivities against *An*. *stephensi*. These findings are in agreement with that of Chore et al. [41], who suggested that solvent polarities used for extraction could influence the mortality of mosquito vectors.

Crude aqueous leaf extracts of the plants, *M*. *foetida* and *Z*. *scabra* at 100 ppm possessed good larvicidal properties relative to the positive control (Temephos). Similar trends were also observed in the late third to early fourth instar larvae of *An. stephensi* with aqueous leaf extract of *C*. *aurea* at 150 ppm. Equally, methanol extracts of *C*. *aurea* at 150 ppm and *M*. *foetida* and *Z*. *scabra* at 200 ppm caused more than 95% larval mortality. Those concentrations of the extracts which showed potent effect were high. This requires using a large amount of the plants if such extracts have to be applied in vector control operations. Therefore, this limitation of the study has to be considered in future research on botanical insecticides.

Ghosh et al. [26] reported that insecticidal effects of plant extracts can vary due to the differences in plant species, mosquito species, geographical variation, extraction methodology, and polarity of solvents used during extraction. Similarly, the finding of the current study varies among concentrations and extraction solvents. Therefore, in agreement with this study, all aqueous crude leaf extracts proved to be sufficiently effective on larvae of *An*. *stephensi*. Interestingly, the aqueous crude leaf extract of *M*. *foetida* was found to have potent larvicidal activity with low LC_50_ and LC_90_ values of 34.61 and 57.61 ppm, respectively, compared to the other two plant extracts assayed concurrently. In other studies, aqueous extracts of *M*. *foetida* have been shown to have larvicidal activity against *An*. *gambiae* and *An*. *coluzzii* larvae with LC_50_ values of 593.96 and 505.19 ppm, respectively [42]. This high difference in larvicidal activities in the current finding could be due to differences in the mosquito species, extraction methods, locality of the plant, preparation of test concentration, and parts of the plant, which are different from those used in this study.

To our knowledge, previous reports are not available on the insecticidal activity of *Z*. *scabra* crude extracts against mosquito vectors elsewhere. This study demonstrated the effective larvicidal activity from the *Z*. *scabra* aqueous (LC_50_ = 35.85 ppm; LC_90_ = 68.26 ppm), and methanol (LC_50_ = 41.32 ppm; LC_90_ = 99.06 ppm) crude leaf extracts against *An*. *stephensi* larvae, where larvicidal activities also showed higher mortality better results than *C*. *aurea* extracts. Previous studies also demonstrated that extracts of *Z*. *scabra* were found to be effective against stored product insect pest [34]. These indicate that the plant has bioactive secondary metabolites that can be used to control insects. The presence of secondary metabolites claimed to have insecticidal activities such as phenol, tannins, flavonoids, and glycosides have been identified in methanol extract of *Z*. *scabra* leaves in previous studies [43, 44]. Such promising larvicidal potency from this plant against *An*. *stephensi* larvae may be associated with the presence of these active secondary metabolites.

In this study, crude aqueous and methanol extracts of *C*. *aurea* leaf were also shown to have high larvicidal activity against *An*. *stephensi* larvae with LC_50_ and LC_90_ values of (38.69 ppm, 108.28 ppm), and (43.25 ppm, 96.02 ppm), respectively. This result is comparable with the methanol extract of *C*. *aurea* leaf extract, which has shown high larval mortality against *An*. *arabiensis* with LC_50_ and LC_90_ values of 84.85 and 192.29 ppm, respectively, in previous studies [45].

Methanol extract of *M. foetida* leaf showed relatively lower larvicidal efficacy than other test extracts against *An. stephensi* larvae with LC_50_ = 99.50 and LC_90_ = 188.76 ppm, in the present study. However, this result is preferable to methanol *M*. *foetida* extract against the larvae of *An*. *gambiae* and *An*. *coluzzii* with LC_50_ values of 276.32 and 235.31 ppm, respectively [42]. A previous study demonstrated that *M*. *foetida* is known for its insecticidal and repellent activities against mosquitoes [33]. Crude aqueous extract of *M*. *foetida* leaf was shown to have high phenolic and flavonoid secondary metabolites [46], which might have contributed to its larvicidal activity.

The values of LC_50_ and LC_90_ obtained by the larvae in this study are lower than those reported by Dey et al. [47], who found that the maximum larvicidal activity from the *Piper longum* aqueous (LC_50_ = 133.42 ppm; LC_90_ = 279.61 ppm) and methanol (LC_50_ = 134.71 ppm; LC_90_ = 464.73 ppm) crude leaf extracts against *An*. *stephensi*. In another study, Mohankumar et al. [48] showed insecticidal promising from the methanol extract of *Annona reticulate* leaf against *An*. *stephensi* larvae with LC_50_ and LC_90_ values of 262.71 and 636.94 ppm, respectively. Therefore, this study suggests that the three plant species could have a beneficial role in the control of mosquitoes.

Plant extracts can be used either as insecticides for killing larvae or adult mosquitoes or as repellents for protection against mosquito bites, depending on their activity [49]. In this study, the effects of test plant extract on adults of *An*. *stephensi* are remarkably less than those recorded on larvae. The adulticidal activity of *M*. *foetida* crude leaf extracts showed less effectiveness against *An*. *stephensi* and its effects were not significantly different from the control treatment. This low activity might be due to the solvents used to extract the bioactive metabolites, which have lower efficacy against the adults.

In the present study, low LC_50_ values against the adults of *An*. *stephensi* was found from the aqueous (LC_50_ = 176.20 ppm) and methanol (LC_50_ = 205.41 ppm) crude leaf extracts of *Z*. *scabra* compared to other test plant extracts. Previous studies revealed extracts of *Z*. *scabra* have effective insecticidal activities against aphids, glowworms, and mill bugs [50]. It also has been reported that *Z*. *scabra* crude extracts to have anti-plasmodial effects [51].

In contrast, aqueous and methanol extracts of *C*. *aurea* showed less mortality against adults of *An*. *stephensi* with LC_50_ values of 297.75 and 333.28 ppm, respectively. Hiruy and Getu [52] reported potent adulticidal activity from the aqueous, ethanol, and acetone extracts of *C*. *aurea* against adults of maize weevils, agriculturally important insect species. Although the WHO does not specify any criteria for classifying the larvicidal and adulticidal potentialities of plant products, some authors use LC_50_ values as a criterion for activity level determination. Specifically, as noted in other studies, the product is very active if LC_50_ < 50 ppm, the product is active if LC_50_ < 100 ppm, the product is moderate if LC_50_ is between 100 ppm-200 ppm, and the product is weakly effective if LC_50_ is between 200 ppm-750 ppm, while the product is inactive if LC_50_ > 750 ppm [53, 54]. Accordingly, the adulticidal activity of *C*. *aurea* extract could be categorized as weakly effective against *An*. *stephensi*.

## Conclusion

This study showed that crude aqueous and methanol extracts of *C*. *aurea*, *M*. *foetida*, and *Z*. *scabra* leaves could be considered as foreseeable products to be developed as potential larvicides against *An*. *stephensi* larvae. In addition, crude leaf extracts of *C*. *aurea* and *Z*. *scabra* are potential candidate insecticides against the adults. These three plants could be used to develop effective, safe, biodegradable, and cheap botanical insecticides for vector control, potentially leading to improved resistance management targeted against malaria vectors in Ethiopia and elsewhere. Therefore, further chemical analysis studies on the identification, preparation, and formulation of bioactive compounds from plants are essential.

## Data Availability

The datasets used during the current study are available from the corresponding author on reasonable request.

## Abbreviations

Hrs: Hours
IRS: Indoor residual spraying
LC_50_: Lethal Concentration resulting in 50% mortality
LC_90_: Lethal Concentration resulting in 90% mortality
LLINs: Long-lasting insecticidal nets
ppm: Parts per million
SPSS: Statistical Package for the Social Sciences
WHO: World Health Organization
